# Postural control during quiet bipedal standing in rats

**DOI:** 10.1371/journal.pone.0189248

**Published:** 2017-12-15

**Authors:** Tetsuro Funato, Yota Sato, Soichiro Fujiki, Yamato Sato, Shinya Aoi, Kazuo Tsuchiya, Dai Yanagihara

**Affiliations:** 1 Department of Mechanical Engineering and Intelligent Systems, The University of Electro-communications, Chofu, Tokyo, Japan; 2 Department of Life Sciences, The University of Tokyo, Meguro-ku, Tokyo, Japan; 3 Department of General Education, Chiba Institute of Technology, Narashino, Chiba, Japan; 4 Department of Aeronautics and Astronautics, Kyoto University, Kyoto, Japan; Tokai University, JAPAN

## Abstract

The control of bipedal posture in humans is subject to non-ideal conditions such as delayed sensation and heartbeat noise. However, the controller achieves a high level of functionality by utilizing body dynamics dexterously. In order to elucidate the neural mechanism responsible for postural control, the present study made use of an experimental setup involving rats because they have more accessible neural structures. The experimental design requires rats to stand bipedally in order to obtain a water reward placed in a water supplier above them. Their motions can be measured in detail using a motion capture system and a force plate. Rats have the ability to stand bipedally for long durations (over 200 s), allowing for the construction of an experimental environment in which the steady standing motion of rats could be measured. The characteristics of the measured motion were evaluated based on aspects of the rats’ intersegmental coordination and power spectrum density (PSD). These characteristics were compared with those of the human bipedal posture. The intersegmental coordination of the standing rats included two components that were similar to that of standing humans: center of mass and trunk motion. The rats’ PSD showed a peak at approximately 1.8 Hz and the pattern of the PSD under the peak frequency was similar to that of the human PSD. However, the frequencies were five times higher in rats than in humans. Based on the analysis of the rats’ bipedal standing motion, there were some common characteristics between rat and human standing motions. Thus, using standing rats is expected to be a powerful tool to reveal the neural basis of postural control.

## Introduction

Postural control in human bipedal standing is affected by non-ideal elements such as delayed sensation and heartbeat noise. Nevertheless, postural control functions such that the center of mass (COM) is always kept within a small range, is accomplished even in varying environments. To maintain such functionality, studies have shown that the controller utilizes body dynamics [1, 2] or nonlinear control [3, 4]. These mechanisms have been studied in experiments involving humans and dynamical analyses. However, in order to understand the underlying neural organization of postural control and its dysfunction in neurological disorders, an experimental setup with more direct access to the neural system is required. There are various powerful interventional methods to manipulate the neural control system, such as pharmacological neuronal lesions and gene manipulations. However, such invasive approaches in humans are limited. Furthermore, measurement of the standing motion in animals with neural lesions will enable a direct approach by which to study the relationship between neural systems and postural control. Therefore, the development of an animal model to study the mechanism of postural control is advantageous.

Previously, researchers studied muscle coordination for postural control using (quadrupedal) standing cats subjected to disturbances from many directions [5, 6]. Quadrupedal and bipedal standing differ in two aspects, namely the control target and control strategy. In terms of control target, quadrupedal standing can maintain the COM position in a comparatively large range, bounded by four legs, while bipedal standing in a sagittal plane requires the COM to remain within a small range. In terms of control strategy, quadrupedal standing requires control of the balance of the load for each leg, which is not required in bipedal standing in a sagittal plane. Therefore, the measurement of bipedal standing in an animal model is more desired for elucidating its mechanisms.

Monkeys and birds have been previously used as animal models of bipedal motion. Studies using monkeys have reported differences in bipedal and quadrupedal walking (Japanese macaque [7–10], bonobo [11], reviewed in [12]) and the evolution of human walking (chimpanzee [13], reviewed in [14]). Studies using birds have discussed the energetic effect required for the transition from walking to running (guinea fowl [15], emu and ostrich [16], lapwing, oystercatcher, and avocet [17]). Moreover, animal models of neural ataxia, such as monkey models of Parkinson’s disease (transgenic [18, 19], neurotoxin [20], reviewed in [21, 22]), stroke (reviewed in [23]), and spinal cord injury have been investigated. For example, changes in motion during the recovery process of monkeys with spinal cord injury have been reported [24, 25]. However, experiments of bipedal motion using animal models have been restricted to dynamic motions like walking, with no research on bipedal quiet standing. Moreover, the use of monkeys to model neural ataxia is limited in its feasibility and value due to their long-life expectancy and need for extensive training.

In comparison, rodent models enable more detailed analysis of the ataxic neural system. Previously, many rodent models of neural ataxia, such as rodents with stroke [26] and spinal cord injury [27], have been used to investigate the effect of ataxia on motor function, such as Parkinson’s disease [28], stroke [29, 30], and spinal cord injury [31, 32]. The neural structures involved in these conditions have also been evaluated in depth. For example, previous studies have demonstrated neuronal rewiring after stroke [33]. Previous research has also demonstrated that rats can move with their lower legs alone when their upper legs have been amputated [34, 35] or through training [36]. Furthermore, bipedal walking in rats and the change in the trunk angle between bipedal and quadrupedal motions have also been previously investigated [36]. However, since prior research in rats has focused solely on joint motion during walking, little is known about their postural control during quiet standing. The COM in quadrupedal freely moving rats was measured over a short duration [37], but there has been no research on the bipedal motion of quiet standing. Developing an experimental model for measuring bipedal standing in rats would enable the investigation of postural control during quiet standing and its neural basis.

Based on those previous findings, the present study proposes an experimental design using rats for studying the postural control of bipedal standing. The motion of the rat was measured with a motion capture system and floor reaction sensors, and the characteristics of COM and body segments were analyzed. In order to compare the results in rats to those from previous human postural control studies, the frequency characteristics and intersegmental coordination of rat motion were also analyzed in the present study.

## Material and methods

### Experimental setup

**Fig 1:**
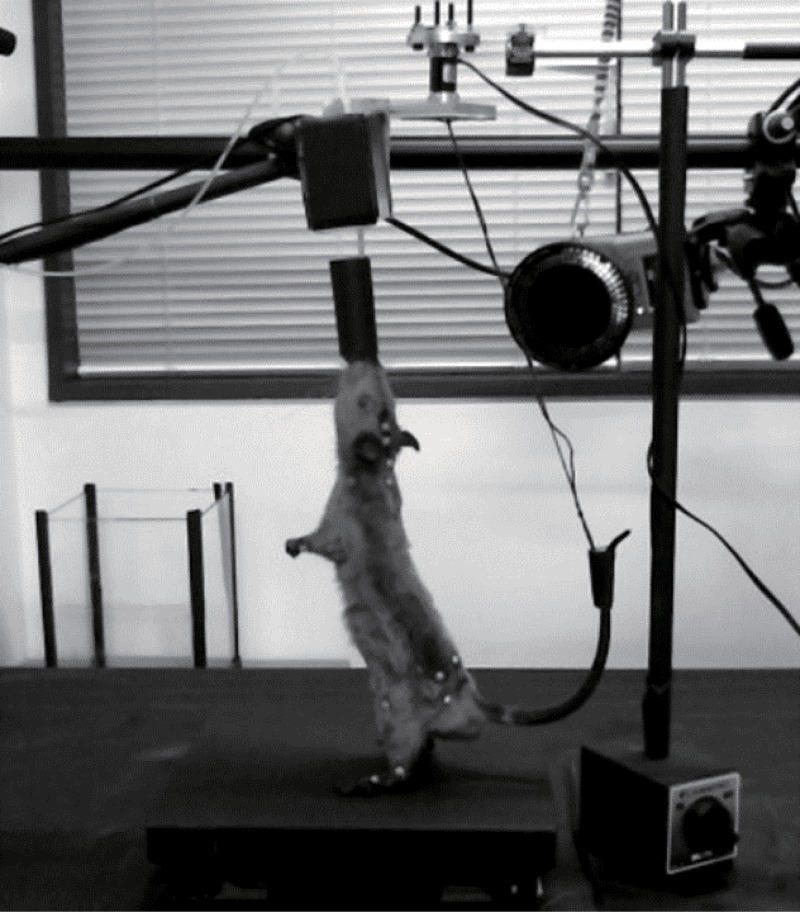
Experimental design. Rats stood on a force plate (230 mm wide and 53 mm high). The rat was surrounded by a motion capture camera. The white circles on the body of the rat were reflective markers for the motion capture system. The black cylinder at the mouth of the rat was a water supply, and the front edge of the water supplying tube was larger than the rat’s mouth to avoid biting. This cylinder was also equipped with a force sensor for measuring the force given to the mouth. The tail was hung by a string and connected to a separate force sensor.

The experimental environment (Fig 1) consisted of a force plate (TF-2020-A, Tec Gihan, Japan) surrounded by a motion capture system (Oqus300+, Qualisys, Sweden). The reward was provided through a water supply system that was placed above the rat at a height that the rat could not reach without standing bipedally. The water supply was composed of a soft tube that does not require the rat to recline while drinking. Moreover, the end of the water supply tube was larger than the mouth of the rat to prevent it from being bitten. The water supply was also equipped with a three-dimensional force sensor (Tec Gihan, Japan) to measure the force applied by the mouth. Rats were encouraged to maintain their standing posture via a water reward provided by a water supply, and this water supply placed at a high position potentially worked for attracting attention of the rats. The rat could move freely on the force plate. The tail was tethered to the environment with a string and did not touch the ground, in order to prevent its use to support the bipedal posture. The string was also connected to a three-dimensional force sensor (Tec Gihan, Japan) to measure the force applied by the tail.

Measurements were started when the observer visually confirmed that the rat was standing bipedally on its hindlimbs. The experiment was considered successful if the rat remained in a standing posture for over 200 s. Reflective markers were attached to the skin overlying 21 body landmarks. The landmarks at the center of the body were as follows: the top of the head (H1), the centers of the right and left scapulae (ShM), one and two thirds of H1 and ShM (H2 and H3), and the center of the right and left iliac crests (HipM). Furthermore, the following body landmarks were placed on the right and left sides of the body: scapulae (ShR and ShL), elbow joint (ElR and ElL), distal head of the ulna (ArmR and ArmL), iliac crest (HipR and HipL), greater trochanter (GtR and GtL), lateral condyle of the knee (KneeR and KneeL), lateral malleolus (AnkleR and AnkleL), and distal end of the fifth metatarsal (MtR and MtL). The marker positions were subsequently measured by using a motion capture system. The ground reaction force was also measured by using the force plate. The sampling rates of the motion capture system and force plate were 500 Hz and 1,000 Hz, respectively. The force on the mouth and tail were also measured using the force sensor with a sampling rate of 500 Hz.

A total of 10 intact Wistar rats, aged 15–16 weeks, with an average weight and body height during bipedal standing of 210 ± 40 g and 175 ± 15 mm, respectively, were used in the current study.

This study was approved by the Ethical Committee for Animal Experiments at the University of Tokyo and conducted in accordance with the Guidelines for Research with Experimental Animals of the University of Tokyo.

### Data processing

Data on the ground reaction force and motion were obtained with the force plate and motion capture system, respectively. Two other force variables were obtained using the force sensors at the mouth and tail, and were used to confirm the effect of force at the mouth and tail on postural control.

The ground reaction force was used to calculate the center of pressure (COP) and investigate the body sway while standing. The human body is known to have a large and slow (less than 1 Hz) sway in its sagittal motion [38–41]. Thus, the body sway in the sagittal plane of the standing rat at a slow frequency range was analyzed. The COP data were filtered using a low-pass filter (2nd-order Butterworth filter) with a cutoff value of 5 Hz only when their time series were displayed.

**Fig 2:**
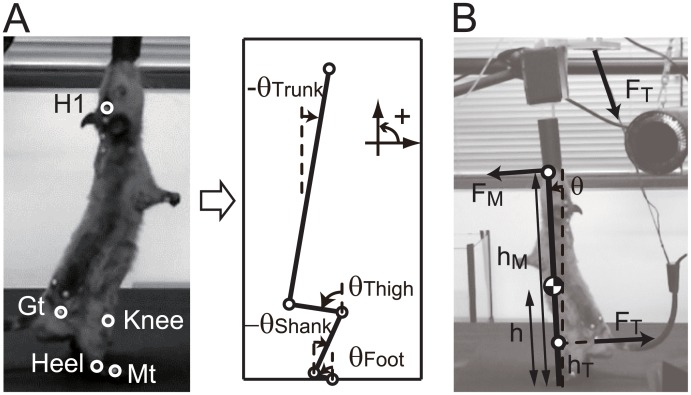
Definition of body angles, force, and length. A: definition of body angles. The body angles were defined by the position of Head (H1), Greater trochanter (GT), Knee, Heel, and Metatarsal (MT) markers measured by the motion capture system. All angles were defined as elevation angles from a vertical line in the sagittal plane. B: definition of the force and length for the calculation of the torque due to external forces.

Motion data were used to analyze the coordination of the body segments and were filtered using a low-pass filter (2nd-order Butterworth filter) with a cutoff value of 10 Hz. The four elevation angles of the foot (θ_Foot_), shank (θ_Shank_), thigh (θ_Thigh_), and trunk (θ_Trunk_) were calculated as shown in Fig 2A using the marker positions of the head (H1), greater trochanter (Gt), knee (Knee), heel (Heel), and metatarsus (Mt). The marker positions were calculated by averaging the right and left sides of the body (e.g., Gt was the average of GtR and GtL). All elevation angles were defined as angles from a vertical line in the sagittal plane.

The COM was also calculated from the motion data, in addition to the positional relationship between the COM of each body segment and the endpoint of the segment. The COM of the segments was obtained after the experiment by using the postmortem bodies of three rats. Briefly, the postmortem rat bodies were separated into body segments, which were hung using two different supporting points. The vertical axis from each supporting point was recorded. In addition, the COM of the body segment was estimated as the crossing point of the two axes, and the position of the estimated COM from the endpoint of the segment was recorded. Finally, the mass of each segment was measured.

Supporting information has been provided to describe the detailed results of the COM parameters. The COM of each segment was calculated using these body parameters and the motion of segment, and the COM of the whole body was calculated using the weighted average of the segmental COM by mass.

### Evaluation of the external force

From the force sensors on the water supply and tether connected to the tail, the effect of the external forces at the mouth and tail on postural control was evaluated. The measured force data were filtered using a low-pass filter with a cutoff value of 5 Hz and high-pass filter with a cutoff value of 0.01 Hz (2nd-order Butterworth filter). In order to evaluate the effect of these values, the torque generated by forces at the mouth (*τ*_M_) and tail (*τ*_T_) was calculated and compared with the torque required for maintaining the posture (*τ*_stable_).

The torque at the mouth and tail was obtained as the product of the length from foot to mouth/tail and force. The length and force are defined in Fig 2B. The length from foot to mouth (*h*_M_) was defined as the distance between the metatarsal marker (Mt) and the head marker (H1), and the length from foot to tail (*h*_T_) was defined as the distance between the metatarsal marker (Mt) and the greater trochanter marker (Gt). The force at the mouth (*F*_M_) was defined as the anterior-posterior direction of the sensor at the mouth, and the force at the tail (*F*_T_) was defined as the net force of the anterior-posterior direction and superior-inferior direction of the sensor at the tail.

In order to estimate the torque required for maintaining the posture, COM motion was modeled with a 1-link inverted pendulum model around the foot.

Jθ¨=mghθ+τ (1)

where *θ* is the angle from the vertical line, *h* is the length from the foot to the COM, *m* is the mass, *g* is the acceleration due to gravity, *J* is the inertia around the foot, and *τ* is the torque around the foot, including the control torque *τ*_control_, torque from the force at the mouth *τ*_M_, and torque from the force at the tail *τ*_T_ (*τ = τ*_control_ + *τ*_M_ + *τ*_T_). The inverted pendulum falls down when the acceleration of the angle cannot be stopped (> 0), and this acceleration value is determined by the relationship between *mghθ* and *τ*. In order to counteract the gravitational torque *mghθ*, the torque around the foot should be equal to or over that value, i.e., the torque required for stable posture is -*mghθ*. This indicates that the effect of *τ*_M_ and *τ*_T_ is estimated by the relationship between *mghθ* and *τ*_M_, and between *mghθ* and *τ*_T_.

The relationship between the torque of the mouth/tail and the torque required for stability was evaluated by determining the amount of supporting torque from the mouth and tail when the posture was most inclined (*θ*_*max*_). For that purpose, the maximum value of the stability torque *mghθ*_*max*_ was calculated, and this value was compared with the maximum values of *τ*_M_ and *τ*_T_. Here, if the torque of the mouth or tail had approximately the same value as *mghθ*_*max*_, postural control around the legs was barely applied, and the motion could not be regarded as bipedal standing; thus, such trials should be excluded. In case the torque of the mouth or tail was small, for example, lower than half of *mghθ*_*max*_, postural control for maintaining body stability would be applied, and the experimental data would be worth analyzing.

The calculation of the length and COM values required motion capture data; therefore, the above-mentioned calculation was performed for the rat with motion capture data. The average (± standard deviation [SD]) parameters of the rat with the motion capture data were as follows: *h*_M_, 166.3 (± 7.1) mm; *h*_T_, 43.1 (± 8.4) mm; *m*, 207.4 (± 15.2) g; and *h*, 112.3 (± 10.1) mm.

### Spectral analysis

The power spectrum density (PSD) was calculated from the measured COP. This study focused on a frequency range from 0.01 to 5 Hz since a characteristic cyclic motion related to nonlinear control was reported to lie in the slow frequency range (< 1 Hz) in human studies [38–41]. In order to calculate the motion in this range, only the data files over 150 s were used for spectral analysis.

The PSD was calculated by using the fast Fourier transform (FFT) and maximum entropy method (MEM). The MEM calculation was performed by using the Burg method, with dimensions at 512 [42, 43]. Since MEM was relatively more robust than FFT in the case of a limited number of data points, the PSD calculated using MEM was used for peak frequency detection. The second-order differential of the PSD was calculated, and its minimum frequency was used as the peak frequency.

### Analysis of intersegmental coordination 1: Extraction of the coordination patterns

Studies on human walking have used principle component analysis (PCA) and singular value decomposition (SVD) for evaluating the intersegmental coordination [44–47]. One study successfully used PCA to extract the intersegmental coordination from the human standing motion [48]. Based on these studies, we calculated the intersegmental coordination from measured data using SVD as follows:

1. The matrix of the segmental motion *Θ*(*θ*, *t*) was composed by arranging the time series of the elevation angles of body segments (θ_Foot_, θ_Shank_, θ_Thigh_, θ_Trunk_) as follows:
  $$\Theta (\theta ,t)=[\begin{array}{ccc} \theta_{Foot}(t_{1}) & \theta_{Shank}(t_{1}) & \cdots \\ \theta_{Foot}(t_{2}) & \theta_{Shank}(t_{2}) & \cdots \\ \vdots & \vdots & \ddots \end{array}]$$ (2)
2. The temporal average of each column of the matrix *Θ*(*θ*, *t*): *Θ*_0_(*θ*) was calculated and *Θ*_0_(*θ*) was subtracted from *Θ*(*θ*, *t*). The *Θ*_0_(*θ*) represented time-invariant posture (average posture) and *Θ*(*θ*, *t*)-*Θ*_0_(*θ*)|_*t*_ represented the motion from the average posture. The *Θ*_0_(*θ*)|_*t*_ was a matrix that possessed *Θ*_0_(*θ*) for *t* times in the row direction.
3. The SVD was performed as follows:
  $$\Theta \left(\theta ,t\right)=\Theta_{0}\left(\theta \right){|}_{t}+\sum_{i}\lambda_{i}v_{i}z_{i}^{\text{T}}$$ (3)

where *λ*_*i*_, *ν*_*i*_, and *z*_*i*_ are a singular value, a column vector of the left singular matrix, and a column vector of the right singular matrix, respectively.

Through the above-mentioned analysis, a group of segments with high correlation (intersegmental coordination) was obtained as *z*_*i*_, while the time series of the group was obtained as *ν*_*i*_. Moreover, the number of coordinations that composed the standing motion could be calculated as the contribution ratio (*R*_*i*_) from a singular value (*λ*_*i*_). For example, the motion extent of the first intersegmental coordination (*z*_1_) included in the standing motion was *R*_1_ = *λ*_1_^2^/(Σ *λ*_i_^2^), and the motion extent of the first and second intersegmental coordinations *z*_1_ and *z*_2_ included in the standing motion was *R*_2_ = (*λ*_1_^2^+*λ*_2_^2^)/(Σ *λ*_i_^2^) [46].

### Analysis of intersegmental coordination 2: Identification of the physical meaning of the coordination patterns

Previous human studies have indicated that the standing motion is composed of coordinative motion reflected in the COM motion [48] or in in-phase and anti-phase motions [49]. The extracted intersegmental coordination possibly corresponded to these characteristic motions. In order to validate them numerically, motion generated by the intersegmental coordination was compared with physically important motion [47].

The intersegmental coordination (*z*_*i*_) was composed of a ratio among segmental angles. Thus, for the sake of comparison, the characteristic motion during standing, such as change in COM position, COM angle, and trunk angle, was represented in the same manner, i.e., as the ratio among segmental angles. Representing the angle in this manner was simple, in such a way that the trunk angle was represented as follows:

[0,0,0,1]T (4)

The correlation coefficient between this matrix and intersegmental coordination (*z*_*i*_) can represent the extent of *z*_*i*_ that is composed of trunk motion. The above-mentioned matrix for the evaluation has the relationship between segmental angles *Θ* = [*θ*_*Foot*_, *θ*_*Shank*_, *θ*_*Thigh*_, *θ*_*Trunk*_]^T^ and trunk motion *r* as follows:

r=[0,0,0,1]Θ, (5)

where the matrix works for extracting the trunk motion from the whole-body motion.

We subsequently considered the relationship to involve the position instead of the angle. If small movements of segmental angles *ΔΘ* changed the position of a certain point in the body, such as COM, for *Δr*, the relationship can be written as follows:

Δr=JTΔΘ (6)

This matrix, *J*, can be obtained as the partial differentiation of *r* by *Θ*, and is called the Jacobian matrix (see Supporting Information for more details on its calculation). By using the Jacobian matrix, the characteristic motion can be represented by a ratio among segmental angles similar to the intersegmental coordination. In this study, the Jacobian matrix of COM and trunk motions were calculated and compared with the intersegmental coordination using the cosine correlation. As a result, the extent of intersegmental coordination that is composed of each characteristic motion can be numerically evaluated.

## Results

### Experimental data

A total of 10 rats successfully stood bipedally for 1–5 trials (Table 1). A successful experiment was defined as one in which steady standing for longer than 200 s occurred. After the experiments, data were carefully separated according to the standing condition of the rats, and only continuous state data were extracted. Standing durations were determined by an analyst who observed the motion of the rats via video.

**Table 1:** List of rats, trials, duration (Dur), and obtained data types. Data type "K" represents the trials that measured the rat’s motion using the motion capture system. Data type "F" represents the trials used for frequency analysis. The Dur was measured in seconds (s).

	Dur (s)	Type		Dur (s)	Type		Dur (s)	Type
Rat1	360.0	K/F	Rat4	220.0	K/F	Rat8	363.9	F
Rat1	174.3	K/F	Rat4	100.0	K	Rat8	361.9	F
Rat2	165.4	K/F	Rat5	102.0	K	Rat8	280.3	F
Rat3	63.0	K	Rat6	78.0	K	Rat8	361.3	F
Rat3	112.0	K	Rat7	266.5	F	Rat8	202.8	F
Rat3	135.0	K	Rat7	279.9	F	Rat9	362.2	F
Rat3	90.6	K	Rat7	332.2	F	Rat10	206.9	F

The duration of continuous standing, for 21 trials in total, had a maximum and minimum of 363.9 s and 63 s, respectively (Table 1). Both the floor reaction force and motion capture data were obtained for six of the 10 rats (Rat1-Rat6), while only the floor reaction force was obtained for the remaining four rats (Rat7-Rat10). The floor reaction force was used for the frequency analysis of the body sway. We focused on the slow characteristic frequency (i.e., 0.01–5 Hz); thus, only data with duration over 150 s were used for the frequency analysis. In order to classify the data, trials for motion capture data were displayed as "K," while those used for the frequency analysis were displayed as "F" in Table 1.

**Fig 3:**
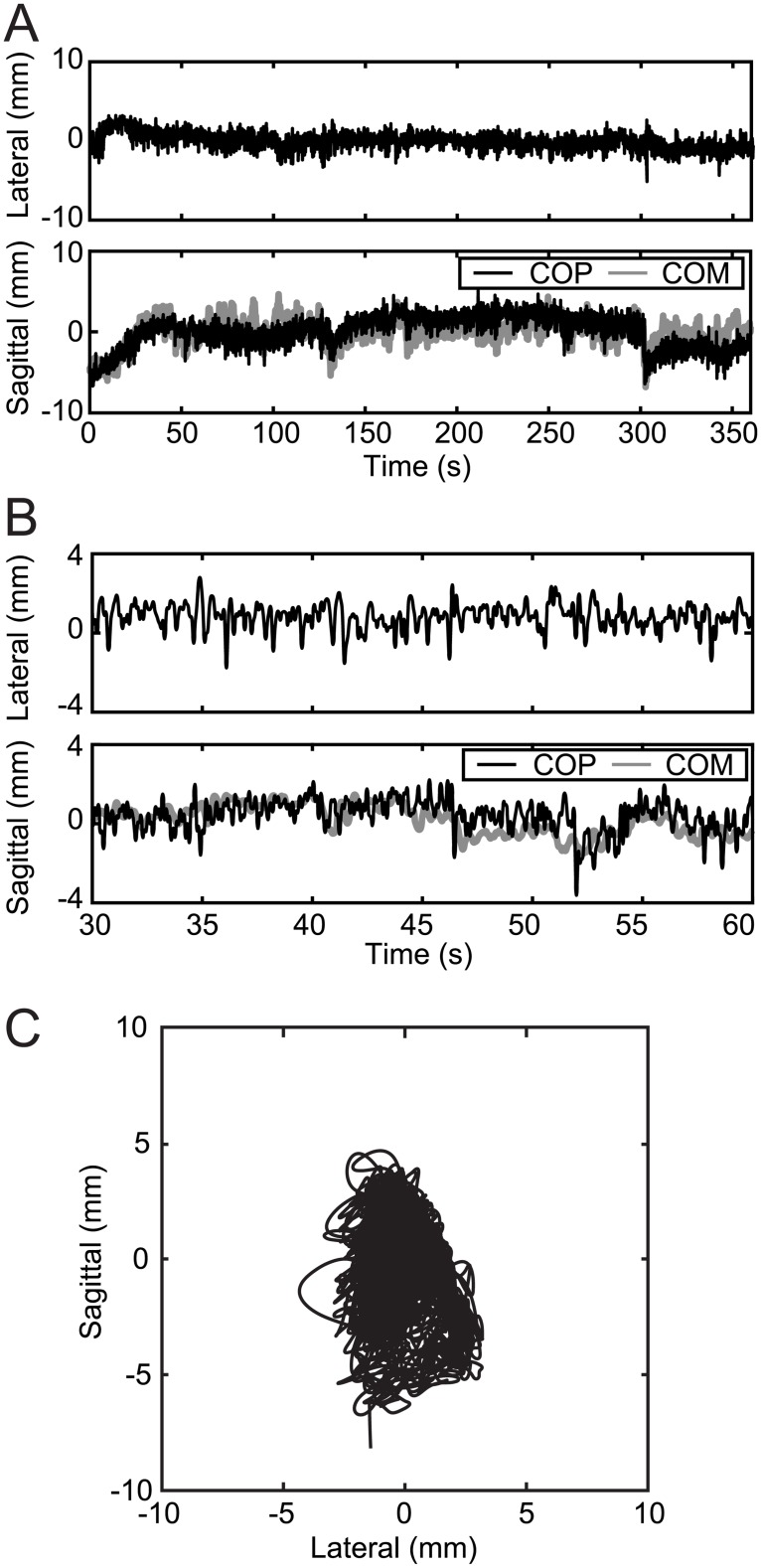
Measured body sway of the standing rat. Data collected were the center of pressure (COP) obtained by the force plate. Representative data from one trial of one rat (Rat 1). A: The time series of COP in the sagittal and lateral direction. The sagittal COP motion (black line) is displayed with the horizontal center of mass (COM) motion (gray line). B: Same time series with (A) in a limited time. The displayed time is 30 to 60 seconds (s). C: The movement of the COP in the horizontal plane.

**Fig 4:**
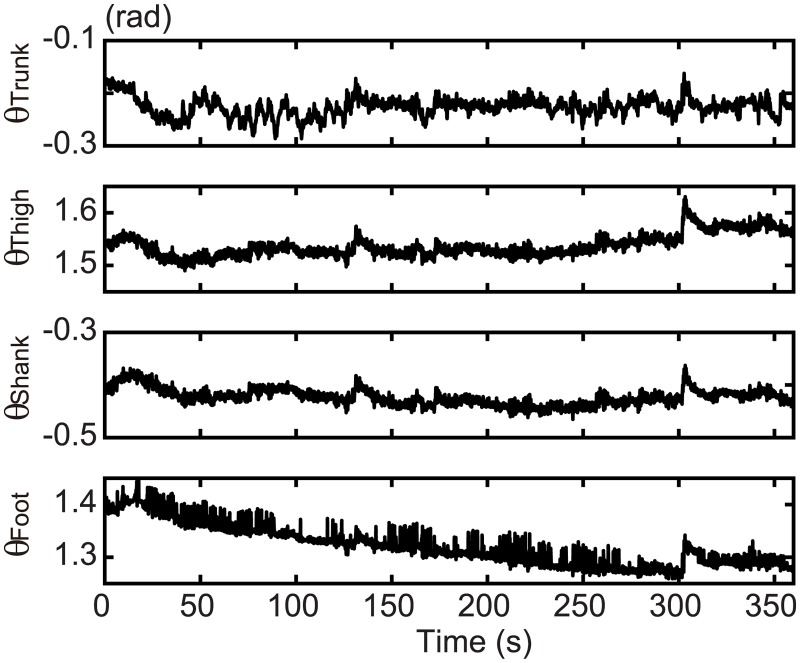
Angular motion of the body segments. Each angle was calculated from the motion capture data as defined in Fig 2A. Representative data from one trial of one rat (Rat 1).

The COP was calculated from the measured floor reaction force (Fig 3), where the rats stood with a continuous body sway of 5–10 mm. To confirm their similarity, the sagittal COP was displayed together with the COM in Fig 3A and 3B. The SD of the COP of each trial is shown in Table 2. The average (± SD) of the SD of the COP motion was 3.29 (± 0.87) mm and 3.04 (± 0.63) mm for the sagittal and lateral directions, respectively. The human body sway in the sagittal plane was approximately 20 mm [41] with an SD of 6.02 (± 1.64) mm. The body sway of rats in the sagittal plane was greater than half of this value. In the current study, the length from the floor to the COM of the rats was about 1/10 that of a human. Therefore, the body sway in rats, in relation to their body size, was large compared to that in humans. The angles of the body segments were calculated using motion capture data (Fig 4). Each segment moved slowly with small fluctuations. The amplitude of the fluctuation was almost similar for every segment.

**Table 2:** Standard deviation (SD) of the center of pressure (COP). The COP was calculated from force plate data, and the magnitude of motion of the COP was evaluated by the SD of the time series of COP motion. Lat, lateral; Sag, sagittal.

	Sag (mm)	Lat (mm)		Sag (mm)	Lat (mm)		Sag (mm)	Lat (mm)
Rat1	2.98	2.20	Rat4	2.73	2.62	Rat8	4.67	4.68
Rat1	2.49	3.44	Rat4	2.51	2.28	Rat8	4.38	3.70
Rat2	2.72	2.69	Rat5	2.74	2.83	Rat8	3.86	2.77
Rat3	2.52	2.43	Rat6	2.86	2.83	Rat8	3.67	3.36
Rat3	3.16	2.52	Rat7	3.75	2.64	Rat8	3.24	2.86
Rat3	2.56	2.52	Rat7	3.18	3.66	Rat9	3.80	4.14
Rat3	2.52	3.31	Rat7	2.83	3.05	Rat10	5.89	3.29

The maximum forces given to the mouth and tail are presented in Table 3A. Forces applied to the rat due to gravity, *mg*, which was the product of mass *m* and acceleration due to gravity *g*, was 2,032.5 (± 149.3) mN, and the values in the table are less than 6% of *mg*. The important data point in this analysis was the effect of these forces on postural control, which was determined by the relationship between the torque generated by these forces and the torque for maintaining the posture. The torque generated by the forces at the mouth and tail around the foot (*τ*_M_, *τ*_T_) was calculated and compared with *mghθ*. The torque generated at the mouth and tail at the most inclined posture (maximum torque), and the torque required for stability are presented in Table 3B. The ratio of the required torque and the torque at the mouth (100*τ*_M_/*mghθ*) and tail (100*τ*_T_/*mghθ*) are also listed in Table 3B. At maximum, approximately 50% of the required torque were applied to the mouth, and 10% were applied at the tail.

**Table 3:** Effect of the forces at the mouth and tail. A: measured maximum forces at the mouth and tail. B: torque generated by the forces at the mouth and tail. The torque of the mouth (*τ*_M_) and tail (*τ*_T_) were calculated as the product of maximum force and the length from foot to mouth and tail. Furthermore, *mghθ*_*max*_ was the torque required to maintain stability in the most inclined posture *θ*_*max*_. The torque ratio is the ratio between the torque generated by the force at the mouth or tail and the torque required for stable standing (100*τ*_M_/*mghθ*_*max*_ and 100*τ*_T_/*mghθ*_*max*_, respectively).

	A		B				
	Maximum Force (mN)		Maximum Torque (mN m)			Torque Ratio (%)	
	Mouth *F*_*M*_	Tail *F*_*T*_	Mouth *τ*_M_	Tail *τ*_T_	*mghθ*_*max*_	Mouth	Tail
Rat 1	104.9	61.3	17.9	2.6	36.6	48.9	7.1
Rat 1	99.4	37.5	17.6	1.6	46.6	37.8	3.4
Rat 2	62.7	58.4	10.1	2.9	45.9	22.1	6.4
Rat 3	58.7	37.4	9.5	1.4	32.4	29.3	4.3
Rat 3	60.7	29.6	10.6	1.4	36.1	29.5	3.9
Rat 3	22.6	41.7	3.7	2.1	54.0	6.9	3.9
Rat 3	40.2	60.0	6.4	1.7	40.2	16.0	4.2
Rat 4	20.0	24.9	2.2	0.8	22.7	9.7	3.4
Rat 4	30.8	14.2	5.2	0.9	48.4	10.8	1.9
Rat 5	90.1	28.9	11.3	1.4	43.1	26.2	3.2
Rat 6	42.4	54.5	7.3	2.8	21.3	34.5	13.0

### Frequency analysis

**Fig 5:**
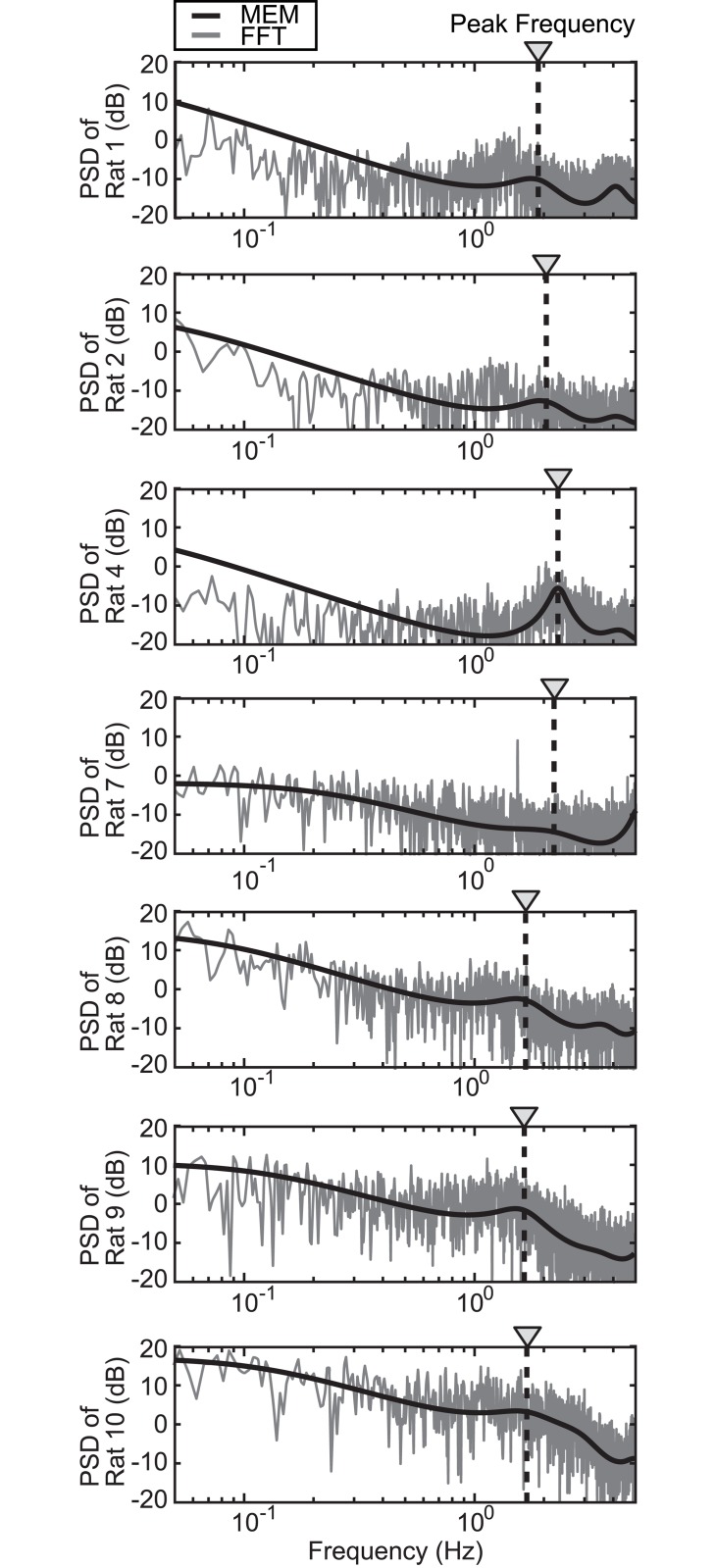
Power spectrum density (PSD) of the body sway. Gray and black curved lines represent PSD values obtained by fast Fourier transform (FFT) and maximum entropy method (MEM), respectively. Black dotted lines represent peak frequencies obtained from the inflection point of MEM. Each data point is a result of one trial of each rat.

The PSD of the COP in the sagittal motion was calculated using the FFT and MEM (Fig 5). The peak frequencies were similar in all 14 trials. The average (± SD) peak frequency was 1.80 (± 0.28) Hz. A previous study demonstrated that the peak frequency of human standing was 0.34 (± 0.04) Hz [41]. Thus, the peak frequency of rats was approximately five times higher than that of humans.

**Fig 6:**
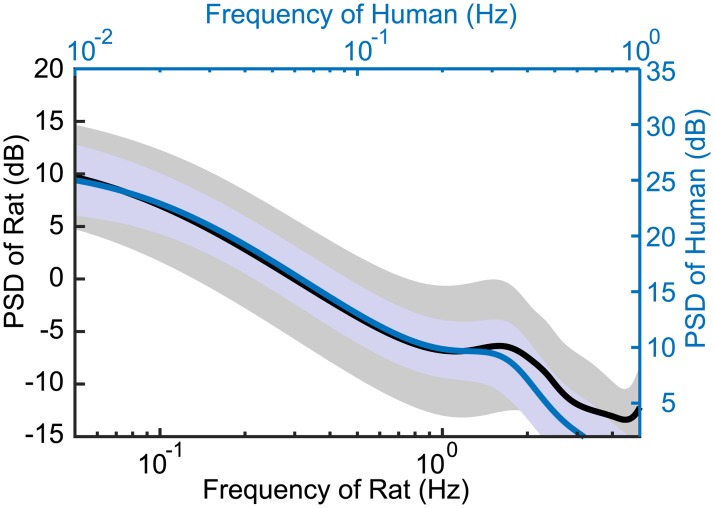
Comparison of power spectrum density (PSD) between rats and humans. The black curved line and area are the average and standard deviation of the values calculated using the maximum entropy method (MEM) for all rats. The blue curved line and area depict data from humans. Black and blue vertical and horizontal axes depict data for rats and humans, respectively. Human data are from a previous study [41].

When the rats’ PSD were superimposed on the human PSD by increasing the frequencies by a factor of five, they were comparable (Fig 6). The black curved line (area) in Fig 6 represents the average (± SD) PSD (calculated by MEM) of rats, while the blue curved line (area) represents that of humans [41]. These results demonstrated that the slow human body sway frequency of less than 1 Hz almost matched the rat’s body sway frequency of less than 5 Hz. This also suggested that the mechanism of postural control has commonality between rats and humans.

### Intersegmental coordination

**Fig 7:**
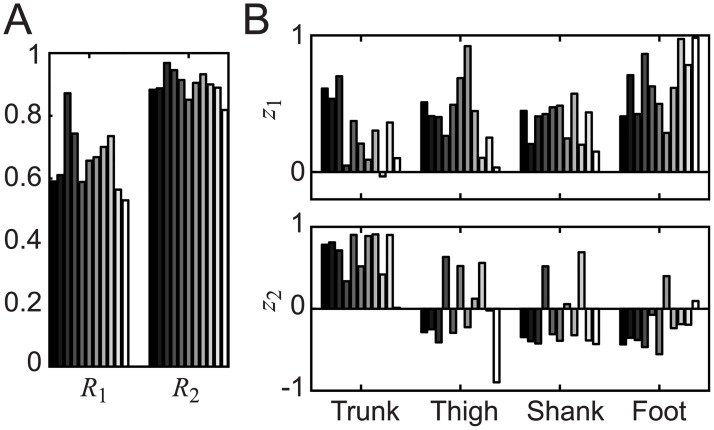
Intersegmental coordination of the standing motion of the rat. Each bar represents the result of a single trial. A: the cumulative contribution ratio. B: the intersegmental coordination. Intersegmental coordination (B) represents a combination of segmental angles that move in a coordinated manner; cumulative contribution ratio (A) represents the extent to which each coordination is included in the standing motion.

The intersegmental coordination was calculated from the elevation angle of the body segments (Fig 7). Each bar in Fig 7 represents the result of each trial. In the results of the cumulative contribution ratio (Fig 7A), the average (± SD) of the cumulative contribution ratio was 0.66 (± 0.10) for one coordination pattern, and 0.90 (± 0.04) for two coordination patterns. The contribution ratio of the other patterns was less than 0.1. In a human study [48], the cumulative contribution ratio was 0.78 for one pattern and 0.90 for two patterns. Therefore, the two rat patterns composed the standing motion similarly to the two human patterns. In order to clarify the number of significant patterns, we calculated the similarity of intersegmental coordination (Fig 7B) among trials. Here, the similarity was measured by cosine correlation, and only the last trial was neglected as outlier. Subsequently, the similarity of the intersegmental coordination among trials was 0.82, 0.55, and 0.09 for the first, second, and third component, respectively. The third component apparently had a large difference among trials, indicating probable noise. Thus, this component was considered less meaningful than the first two components. Based on these results, we evaluated the characteristics of the first and second coordination patterns.

Fig 7B depicts the intersegmental coordination, which represents a set of body segments that move in high correlation to one another. In Fig 7B, almost all segments moved in the same direction in *z*_*1*_, while, in the majority (7 over 11) of the trials, the trunk alone moved in a different direction in *z*_*2*_. Previous research on human standing posture has demonstrated that the standing motion consists of an in-phase mode, where all segments move with the same timing, and an anti-phase mode, where the trunk and legs move with different timing [49]. Our results reflected these human characteristics in rats.

**Fig 8:**
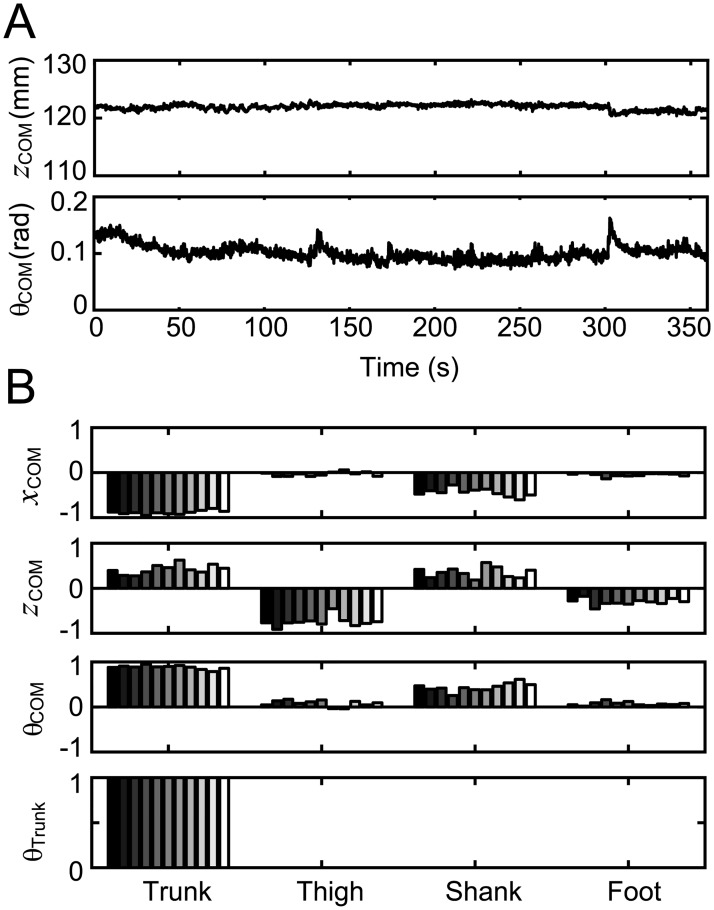
Motion of characteristic points and parts in the standing motion of the rat. Center of mass (COM) motion in the horizontal direction *x*_COM_, vertical direction *z*_COM_, rotation *θ*_COM_, and trunk rotation *θ*_Trunk_. A: A time series of COM motion calculated from segmental motion. Representative data from one trial of one rat. B: The Jacobian matrix of COM and trunk motion. The Jacobian matrix represents the combination of segmental angles that moves the target point or parts, such as the COM or the trunk.

In order to evaluate the physical meaning of each coordination pattern numerically, the intersegmental coordination was compared with the characteristic motion during standing, i.e., the COM motion and trunk motion. The horizontal and vertical position of the COM, and the elevation angle of the COM from the foot were first calculated (Figs 3A and 8A). The Jacobian matrices of the COM and trunk were subsequently calculated as shown in Fig 8B. The Jacobian matrices in the figure represent the combination of segmental angles for changing the COM position horizontally (*x*_com_), vertically (*z*_com_), and rotationally (*θ*_COM_), and for changing the trunk rotationally (*θ*_Trunk_). Finally, the correlation coefficient between the Jacobian matrix and the intersegmental coordination was calculated (Table 4). The values in Table 4 show the correlation between each Jacobian matrix and *z*_*1*_ and *z*_*2*_; the highest values are represented in bold. The results indicated that *z*_*1*_ had the highest correlation with the orientation of the COM for nine of the 11 trials, with values over 0.5 in seven trials and over 0.6 in three trials. On the other hand, *z*_*2*_ had the highest correlation with trunk motion for eight of the 11 trials, with values over 0.5 in eight trials and over 0.7 in seven trials. Above all, the standing motion of rats was found to be composed of two intersegmental coordination patterns that had high correlation with the COM and trunk motion, respectively.

**Table 4:** Correlation coefficients for intersegmental coordination and characteristic motions. The characteristic motions of the horizontal and vertical motion of the COM *x*_COM_, *z*_COM_, rotation of the COM around the foot *θ*_COM_, and trunk rotation *θ*_Trunk_ were compared. Maximum correlation coefficient for each intersegmental coordination (*z*_1_, *z*_2_) are in bold.

Rat	*z*_1_				*z*_2_			
	*x*_COM_	*z*_COM_	*θ*_COM_	*θ*_Trunk_	*x*_COM_	*z*_COM_	*θ*_COM_	*θ*_Trunk_
Rat 1	0.77	0.08	**0.79**	0.61	0.50	0.51	0.49	**0.78**
Rat 1	0.61	0.30	**0.64**	0.54	0.55	0.43	0.54	**0.81**
Rat 2	0.86	0.17	**0.90**	0.70	0.40	0.54	0.35	**0.71**
Rat 3	0.29	0.29	**0.31**	0.05	0.42	0.02	**0.43**	0.34
Rat 3	0.63	0.22	**0.65**	0.37	0.64	0.59	0.63	**0.90**
Rat 3	0.46	**0.54**	**0.54**	0.21	0.31	0.05	0.33	**0.52**
Rat 3	0.18	**0.30**	0.16	0.09	0.87	0.58	0.87	**0.89**
Rat 4	0.52	0.11	**0.54**	0.30	0.64	0.20	0.64	**0.91**
Rat 4	0.11	**0.38**	0.16	0.03	0.74	0.07	**0.78**	0.42
Rat 5	0.58	0.08	**0.61**	0.36	0.48	0.45	0.46	**0.90**
Rat 6	**0.24**	0.21	**0.24**	0.10	0.27	**0.47**	-0.28	0.01

## Discussion

In order to understand the neural mechanism of postural control, we developed an experimental design involving bipedal standing rats, and evaluated the motion of the standing rats in terms of frequency and intersegmental coordination. The present study yielded the following three achievements: (1) an experimental environment that enabled the bipedal standing of rats for long durations (over 200 s) was established; (2) frequency analysis of rat motion demonstrated a slow sagittal body sway with a peak at approximately 1.8 Hz, and the PSD of less than 5 Hz had approximately the same pattern as in human standing motion of less than 1 Hz; and (3) the intersegmental coordination of standing rats demonstrated that the standing motion was composed of two coordination patterns, which corresponded to the COM and trunk motion. In the following discussion, we will first identify the limitations and the validity of the present standing experiment. We will subsequently discuss the comparison between the characteristics of frequency and intersegmental coordination in bipedally standing rats with previous human studies. Finally, the relationship between the proposed method and the mechanism of neural ataxia will be discussed.

### Limitations of the developed experimental environment

The experimental system included a difference from human bipedal standing in that the mouth and tail were touching an external system. A water supply at the mouth was used to provide water as a reward for standing. In order to reduce the effect of the force at the mouth, the water supply tube was larger than the rat’s mouth to prevent biting, and the supply tube was soft to prevent the rat from leaning on it. Despite these measures, the force at the mouth and tail in part supported the rat’s posture, and the forces were thus numerically evaluated.

From the measured forces at the mouth and tail (Table 3A), the forces given to the rats were small. However, the torque calculated from the forces was not small; approximately half of the torque necessary for maintaining the standing posture was produced at the mouth at maximum (Table 3B). Thus, the force at the mouth should not be ignored. Another problem of the external force at the mouth and tail was the sensation of touching. It has been previously demonstrated that a light touch (i.e., force less than 1 N) decreased the sway of the human body during standing [50]. Although a light touch has almost no mechanical effect on the system, it affects the sensory feedback [51].

Time series of the results (Figs 3 and 4) also revealed some different characteristics from the human standing posture. The COM and COP of the first 20 s in Fig 3A and the foot angle in Fig 4 have some trends. The magnitude of these trends was no more than 5 degrees, and thus did not affect the stability. Nevertheless, the quality of the experimental data was certainly less than that of human studies.

The effect of the difference in the skeletal structure between rats and humans on the preservation of the bipedal posture should not be ignored. During standing, the segments of the human body are connected quasi lineally, while the rat’s body segments are flexed. In linear connections, the bipedal posture can be maintained with no torque or at least with a small torque, while the flexed configuration of the rat’s leg always requires a certain amount of control torque.

These limitations imply a possible difference between standing in rats in the present study and free bipedal standing in humans. However, this does not signify that the present standing experiment has no connection to postural control in free bipedal standing. Since bipedal standing in rats is fundamentally unstable, stabilization by a neural control system is necessary. The COM and trunk motion in standing reflect the characteristics of postural control, which may enable us to elucidate its neural basis. If common control strategies exist in rats and humans, understanding the rat controller may elucidate the mechanism of the human controller.

### Relationship between the characteristics of the rat’s motion and the postural control model

In the present study, we analyzed the frequency characteristic and intersegmental coordination of rat motion. Here, by reviewing the experimental bases used for modeling human postural control models, the relationship between the obtained characteristics and postural control models are discussed.

Studies on human standing posture have modeled the body dynamics by simple 1-link or 2-link inverted pendulum models and with feedback control for maintaining the posture. Although the link models with an inverted pendulum provide only an approximation of multiple human joints, they do capture the functionally important motions during standing. The 1-link inverted pendulum models the COM motion, and the 2-link inverted pendulum models the trunk and lower body. Studies on motion analysis have shown that humans move in a coordinated manner [49], and that these coordinated patterns mainly move the COM and trunk. One study extracted the correlating motions from the trunk, thigh, and shank during standing and showed that two coordinated motions could be numerically extracted, and that they corresponded to the COM motion [48]. Therefore, calculating the coordination of motions enables the numerical evaluation of how well the body motion during quiet standing is approximated by 1-link or 2-link inverted pendulum models.

The control of the human standing posture consists of stiffness control, which uses the physical stiffness of muscles and the reflection-induced increase of muscle stiffness [52], and sensory feedback control with sensation delay [53, 54]. The proportional-derivative (PD) or proportional-integral-derivative (PID) control models of feedback control [55–57] have been reported to explain various human standing characteristics, including the precedence of muscle activity over joint motion [58] and the long latency responses indicated by stabilogram diffusion analysis [59, 60]. However, linear PD or PID control with heartbeat noise [61] was reported to be unable to generate the magnitude of body sway during standing [62]. Thus, the existence of nonlinear control, such as no control state [1, 2] or ballistic control [38, 63], have been indicated. Body sway includes a slow periodic motion of less than 1 Hz, and the generation mechanism of periodic motion has been explained as being an effect of nonlinear control [38–41]. Therefore, by evaluating the frequency characteristics of the motion and by investigating the existence of a slow frequency component, the existence of nonlinear control in posture control can be evaluated.

Based on these notions, the following subsections discuss the estimated characteristic of the link body from the characteristic of intersegmental coordination and the existence of nonlinear control from frequency characteristics.

### Intersegmental coordination and estimation of the body model

The required complexity of the body link-model can be determined by considering how to move multiple body segments during standing. If the whole body segments center at one point at the same rate, the body motion can be modelled with 1-link inverted pendulum; if there are two center points, the model becomes the 2-link inverted pendulum. Thus, the number of movement types and their physical meanings are important.

Human bipedal standing is composed of a movement centered at the ankle and hip [64]. Both ankle-centered and hip-centered movements are not a single joint motion, but are composed of coordinated movements of the trunk and leg joint with the same timing (in-phase mode) and different timing (anti-phase mode), respectively [49]. In order to evaluate the coordination of the whole body quantitatively, Pinter et al. [48] performed a PCA on standing motion, and demonstrated that it was composed of two coordination patterns. They also indicated that these coordinations were composed of the COM motion and its reaction. We performed a similar statistical analysis for the whole body segmental motion of standing rats, and showed that the intersegmental coordination of rats correlated with the COM motion corresponding to the in-phase mode and trunk motion corresponding to the anti-phase mode. Thus, the coordination of the rats’ body segments has characteristics similar to human standing. This similarity is not trivial if we focus on differences in the body and skeletal structure between humans and rats. Therefore, these results demonstrated that similar strategies for controlling multi-segments are used by both rats and humans, and that 1-link or 2-link inverted pendulum model may enable modeling the rat body.

### Frequency characteristics and estimation of the control model

Human standing is accompanied by approximately 20 mm of body sway, which is a characteristic of postural control during standing. The body sway has been reported to show a slow peak frequency at approximately 0.4 Hz [65–68]. The present study demonstrated that the standing motion of rats had a peak frequency at 1.8 Hz, and the spectral pattern under that frequency was almost identical to humans except that the frequencies were five times higher in rats. This finding implies that the frequency characteristics of a slow range are generated by a similar mechanism; thus, nonlinear control exists in rats similarly to humans. We subsequently addressed how the peak frequency was generated and the meaning of the displacement between rats and humans.

The frequency characteristic of motion is affected by both the characteristic frequency of control and the time constant of the body. In particular, the 0.4 Hz of human sway was found to be a ballistic state [38, 39] due to a lack of control state in postural control [1, 2]. Thus, the frequency would be largely affected by a diffusion time constant of the body. The diffusion time constants of rats and humans were calculated to test whether they can account for the difference in the characteristic frequency. If the body of a standing rat was modeled by a 1-link inverted pendulum as in Eq (1), the pole (*p*) of this motion would be (*mgh/J*)^*1/2*^ and the time constant would be 1/*p*. We substituted the appropriate values for the rat body and human body [41], i.e., *h* = 0.11 m, *m* = 0.21 kg, *J* = 0.0026 kgm^2^ in rats, and *h* = 0.93 m, *m* = 63.6 kg, and *J* = 56.1 kgm^2^ in humans. The time constants of rats and humans were 0.11 and 0.31, respectively, indicating that the rats’ time constant was approximately 1/3 that of humans, indicating that rats fall three times faster than humans. The characteristic frequency due to a fall was approximately three times higher in rats. These findings demonstrated, in part, the reason why the frequency in rats was higher than that in humans (even though the value was three and not five times higher). Therefore, the difference in the frequency observed in the rats can be partially attributed to differences in their bodies. Furthermore, it also supports the possibility that similar mechanisms yield the peak frequency in rats and humans.

### Future approaches to postural control

The current study succeeded in constructing and measuring bipedal standing in rats over a long duration. Furthermore, we demonstrated a similarity in the frequency and intersegmental coordination pattern between bipedal standing in rats and humans. The coordination of body segments can be changed by several conditions of neural ataxia. For example, the coordination between the knee and other segments decreases in cerebellar ataxia [69]. The model introduced in the present study will enable detailed investigation of the effects of neurological disorders on movement, if ataxic rats are used. Using this analysis model in ataxic rats will reveal the relationship between the state of neural structures, frequency characteristics, and intersegmental coordination. Thus, the bipedal rat model that was introduced in the present study will be an effective tool for elucidating the neural basis of postural control.

## Supporting information

S1 Appendix Measurement of body parameters for COM and calculation of Jacobian matrix. (PDF)
