# Supplementary material for: Postural control during quiet bipedal standing in rats
Source: PLoS One. 2017 Dec 15;12(12):e0189248. doi: 10.1371/journal.pone.0189248 (PMC5731682; doi:10.1371/journal.pone.0189248)
Supplement: S1 Appendix — (PDF) [file pone.0189248.s001.pdf]

Appendix S1

Measurement of body parameters for COM

The center of mass (COM) position of the standing movement was calculated using the relationship between the endpoint of the body segment to the COM for the body segment. This document outlines the method that was used and the measurements of the COM for each body segment.

The COM position was evaluated by measuring amputated body segments of the postmortem rat body as follows:

1. Three postmortem rat bodies were frozen and amputated according to body segments as outlined in S1A Fig.
2. Amputated body segments were hung using two different supporting points, and the vertical axis from each supporting point was recorded.
3. The COM of the body segment was estimated as the crossing point of the two axes, and the position of the estimated COM from the endpoint of the segment was recorded.
4. The mass of each body segment was recorded.

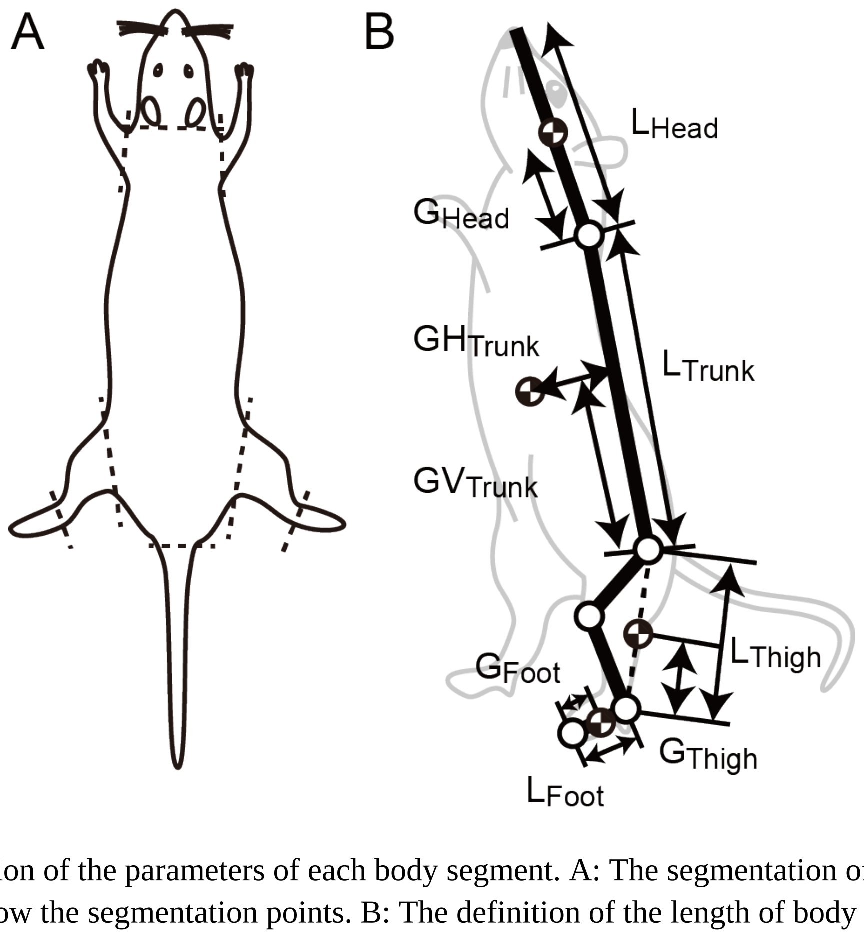

S1 Fig. Definition of the parameters of each body segment. A: The segmentation of the body. Dotted lines show the segmentation points. B: The definition of the length of body segments and the center of mass (COM)

The position of the COM and mass of each segment are outlined in S1B Fig. and S1 Table. The thigh and shank were difficult to separate during the amputation, and their mass was smaller compared to the mass of the whole body. Thus, they were considered a unit. Moreover, when calculating the COM during standing, since the mouth position during standing could not be measured, a marker attached to the top of the head was used to calculate the COM of the head instead of the calculations made to obtain the values in S1 Table.

S1 Table. Body segment parameters of the rat. A: body position of each segment. (R) and (L) represent the right and left sides of the body, respectively. B: body mass of each segment and mass of whole body.

A

	Rat A	Rat B	Rat C	Average
G_{Head}/L_{Head}	0.36	0.43	0.44	0.41
GH_{Trunk}/L_{Trunk}	0.52	0.43	0.51	0.49
GV_{Trunk}/L_{Trunk}	0.33	0.36	0.40	0.36
G_{HT}/L_{HT}	0.50	0.52	0.53	0.52
G_{Thigh}/L_{Thigh} (R)	0.53	0.49	0.56	0.55 (average of R and L)
G_{Thigh}/L_{Thigh} (L)	0.59	0.55	0.62	
G_{Foot}/L_{Foot} (R)	0.623	0.47	0.56	0.55 (average of R and L)
G_{Foot}/L_{Foot} (L)	0.60	0.58	0.47	

B

	Rat A	Rat B	Rat C	Average
Head mass/Total mass	0.08	0.14	0.11	0.11
Trunk mass/Total mass	0.85	0.75	0.79	0.80
Thigh mass/Total mass	0.03	0.06	0.06	0.05
Foot mass/Total mass	0.01	0.02	0.02	0.01
Total body mass (g)	276.0	262.7	256.8	

Calculation of Jacobian matrix

COM position of each segment on anterior-posterior direction is calculated from the length of foot L_{Foot}, shank L_{Shank}, thigh L_{Thigh} and trunk L_{Trunk}, and COM position ratio obtained in the previous section $R_F=G_{Foot}/L_{Foot}$, $R_S=G_{Shank}/L_{Shank}$, $R_{Th}=G_{Thigh}/L_{Thigh}$, $R_{Tr}=G_{HT}/L_{HT}$, $R_F=G_{Foot}/L_{Foot}$, $R_S=G_{Shank}/L_{Shank}$, $R_{Th}=G_{Thigh}/L_{Thigh}$, $R_{Tr}=G_{HT}/L_{HT}$ as follows.

$$\begin{cases} COM_{Foot} = L_{Foot}R_F\cos\left(\theta_{Foot}+\frac{\pi}{2}\right) \\ COM_{Shank} = L_{Foot}\cos\left(\theta_{Foot}+\frac{\pi}{2}\right)+L_{Shank}R_S\cos\left(\theta_{Shannk}+\frac{\pi}{2}\right) \\ COM_{Thigh} = L_{Foot}\cos\left(\theta_{Foot}+\frac{\pi}{2}\right)+L_{Shank}\cos\left(\theta_{Shannk}+\frac{\pi}{2}\right) \\ \qquad +L_{Thigh}R_{Th}\cos\left(\theta_{Thigh}+\frac{\pi}{2}\right) \\ COM_{Trunk} = L_{Foot}\cos\left(\theta_{Foot}+\frac{\pi}{2}\right)+L_{Shank}\cos\left(\theta_{Shannk}+\frac{\pi}{2}\right) \\ \qquad +L_{Thigh}\cos\left(\theta_{Thigh}+\frac{\pi}{2}\right)+L_{Trunk}R_{Tr}\cos\left(\theta_{Trunk}+\frac{\pi}{2}\right) \end{cases} \quad (S1)$$

Then, from these equations and mass ratio m_{Foot}, m_{Shank}, m_{Thigh}, m_{Trunk}, COM of whole body can be obtained as following.

$$\begin{aligned} COM &= m_{Foot}COM_{Foot}+m_{Shank}COM_{Shank}+m_{Thigh}COM_{Thigh}+m_{Trunk}COM_{Trunk} \\ &= \left(m_{Foot}R_F+m_{Shank}+m_{Thigh}+m_{Trunk}\right)L_{Foot}\cos\left(\theta_{Foot}+\frac{\pi}{2}\right) \\ &+\left(m_{Shank}R_S+m_{Thigh}+m_{Trunk}\right)L_{Shank}\cos\left(\theta_{Shank}+\frac{\pi}{2}\right) \\ &+\left(m_{Thigh}R_{Th}+m_{Trunk}\right)L_{Thigh}\cos\left(\theta_{Thigh}+\frac{\pi}{2}\right) \\ &+m_{Trunk}R_{Tr}L_{Trunk}\cos\left(\theta_{Trunk}+\frac{\pi}{2}\right) \\ &= F_F\cos\left(\theta_{Foot}+\frac{\pi}{2}\right)+F_s\cos\left(\theta_{Shank}+\frac{\pi}{2}\right)+F_{Th}\cos\left(\theta_{Thigh}+\frac{\pi}{2}\right)+F_{Tr}\cos\left(\theta_{Trunk}+\frac{\pi}{2}\right) \end{aligned} \quad (S2)$$

By calculating the partial differential equation by Θ for both members, Jacobian matrix becomes

$$\left[-\sin\left(\theta_{Foot}+\frac{\pi}{2}\right)F_F \quad -\sin\left(\theta_{shank}+\frac{\pi}{2}\right)F_s \quad -\sin\left(\theta_{Thigh}+\frac{\pi}{2}\right)F_{Th} \quad -\sin\left(\theta_{Trunk}+\frac{\pi}{2}\right)F_{Tr}\right]^{\mathrm{T}}. \quad (S3)$$

Each angle in the Jacobian matrix represents a standard posture, i.e., the angles of posture that determine from what posture a small change by the differential is considered. In the present research, standard posture is defined as the average posture during standing, which means the average values of each angle is substituted to θ_{Foot}, θ_{Shank}, θ_{Thigh}, θ_{Trunk} for calculating the Jacobian matrix. The COM position ratio R_S is set to 0.5, namely the COM of the shank is regarded to be in the center of the shank. COM position of the superior-inferior direction and angles can be obtained by a similar calculation.
